# Low-Dimensional Palladium Nanostructures for Fast and Reliable Hydrogen Gas Detection

**DOI:** 10.3390/s110100825

**Published:** 2011-01-13

**Authors:** Jin-Seo Noh, Jun Min Lee, Wooyoung Lee

**Affiliations:** Department of Materials Science and Engineering, Yonsei University, Seoul 120-749, Korea; E-Mails: jinseonoh@yonsei.ac.kr (J.-S.N.); junmin@yonsei.ac.kr (J.M.L.)

**Keywords:** Pd nanostructures, hydrogen sensors

## Abstract

Palladium (Pd) has received attention as an ideal hydrogen sensor material due to its properties such as high sensitivity and selectivity to hydrogen gas, fast response, and operability at room temperature. Interestingly, various Pd nanostructures that have been realized by recent developments in nanotechnologies are known to show better performance than bulk Pd. This review highlights the characteristic properties, issues, and their possible solutions of hydrogen sensors based on the low-dimensional Pd nanostructures with more emphasis on Pd thin films and Pd nanowires. The finite size effects, relative strengths and weaknesses of the respective Pd nanostructures are discussed in terms of performance, manufacturability, and practical applicability.

## 1. Introduction

Hydrogen gas (H_2_) has attracted a gradually increasing attention as a new energy source, keeping pace with the needs to cope with the anticipated exhaustion of traditional fossil fuels and to alleviate a global warming issue by reducing the use of fossil fuel [1]. Advantages of hydrogen gas lie in its cleanliness, chemical reactivity, recyclability, and natural abundance. However, hydrogen is highly flammable and becomes explosive when its concentration exceeds 4% in air [2]. For this reason, fast and reliable detection of hydrogen gas is required for the wide-spread realization of H_2_-based applications.

Various approaches have been made to fabricate hydrogen sensors with high sensitivity and fast response [3–11], and some of the initial efforts are typified by the already commercialized hot wire type [12] and electrochemical type [13] sensors. However, those sensors in their early stage of development suffered from drawbacks such as high power consumption, poor hydrogen selectivity, and high operating temperatures. A metal-oxide-semiconductor (MOS) sensor as another type of hydrogen sensor was proposed by Lundström *et al*., which could detect low concentrations of hydrogen gas [14–19]. This sensor, however, showed weak points such as premature saturation of detectable hydrogen concentrations and low sensitivity. More recently, delicate devices such as metal-oxide-semiconductor field-effect transistors (MOSFETs), and particularly high electron mobility transistors (HEMTs) and metal-semiconductor Schottky diodes, have emerged for high performance H_2_ sensing [20–25]. Although these devices have achieved much improved sensitivity and reasonable response times and detection limits, the fabrication processes are complicated, making the devices costly. This is because semiconductors incorporated in the structures are generally III–V compounds such as GaN, InGaP, and AlGaAs or II–VI compounds such as ZnO, which are all hard to grow epitaxially with limited growth areas, and many fabrication steps must be carefully controlled to obtain basic device performance along with optimal device design. In these devices, the choice of a catalytic metal is of importance and palladium (Pd) has been the most widely used one for that purpose.

Since the interaction of Pd and hydrogen was comprehensively studied four decades ago [26], the use of Pd has been extensively pursued in fields such as hydrogen storage, hydrogenation of matter, and hydrogen gas sensors [27–35]. Focusing on the hydrogen sensor area, attention has been paid to Pd as a promising H_2_-sensing material, primarily due to its high sensitivity and selectivity towards H_2_ and potential compatibility with the conventional integrated circuit (IC) fabrication process [36–38]. More concretely, key requirements for a material to be a good hydrogen sensor include high sensitivity, fast response, high H_2_ selectivity, room-temperature-operability, good reversibility, low detection limit, and low power consumption. Pd is recognized as a good hydrogen sensor material because it can satisfy most of these key requirements, although its selectivity to hydrogen may change depending on the type of coexisting gases such as CO, CO_2_, and H_2_S [39,40]. Needless to say, it is desirable for a Pd-based sensor to meet those requirements simultaneously, which is very challenging. In particular, the parameters of sensitivity, response time, and detection limit are of critical importance in determining the performance of a sensor, and they need to be considered in association with the sensor structures and H_2_-sensing methods employed.

Representative H_2_-sensing techniques based on Pd can be categorized as electrical, optical, strain, and chemi-mechanical methods, depending on the physical parameters under detection. Electrical and strain methods measure changes in electrical properties (resistance or conductance) and in sample dimensions, respectively. Sensor geometries for these methods are relatively simple. On the other hand, optical method detects a change in optical properties (reflectance or transmittance) caused by absorption of hydrogen atoms, using optical fibers in most cases. This method is safe, since there is no need to use electric signals that may cause electric discharge and remote sensing in hazardous environments is thus possible. However, light sources such as lasers and rather complex detection systems are required for this method, which makes it weak in both portability and cost-effectiveness [41–44]. Finally, chemi-mechanical methods detect some mechanical change of a small sensing structure. Typical are microcantilevers fabricated by micro-electro-mechanical system (MEMS) processes. The surface of the microcantilevers is usually functionalized with Pd thin films. Volume expansion of a Pd film produced by hydrogen absorption bends the microcantilever and this mechanical change is monitored by parameters such as deflection, resonance frequency, and capacitance. Although this method has the advantages of high sensitivity and low power consumption, several issues such as relatively complex sensor fabrication process, additional signal-detecting systems, and generally slow response still need to be tackled [45–48]. As briefly discussed above, all Pd-based H_2_-sensing methods have advantages and disadvantages at the same time. Nonetheless, the electrical method is singled out as a central subject in this article because it may have more advantages than others in many aspects such as overall performance level, simplicity of the sensor structures, possibility of sensor miniaturization, manufacturability of the sensors, and compatibility of sensor fabrication process with the current IC process. Conventional electrically operating hydrogen sensors based on Pd are classified as ‘normally-on’ sensors, since they keep conductive before their resistance increase upon H_2_ exposure. Bulk Pd sensors typically belong to this class.

The recent development of nanotechnologies has fueled the advent of Pd nanostructures such as two-dimensional (2D) Pd thin films [49–53], one-dimensional (1D) Pd nanowires and nanorods [54–58], and even Pd nanoparticles [59–63]. Using these Pd nanostructures, inverse types of ‘on-off’ sensors can be realized, which operate opposite to the conventional hydrogen sensors. In this class of sensors, electrical resistance drops when a sensor material in the initial open state is exposed to H_2_ [64–68], because a certain type of nanoscopic gap is closed by volume expansion of the Pd nanostructure as demonstrated in Pd mesowire arrays by the Penner group [64]. Although this type of inverse on-off phenomenon is interesting enough, most low-dimensional Pd nanostructures still exhibit resistance-increasing behavior with the adsorption of H_2_ as in bulk Pd sensors. However, the H_2_-sensing performance of the Pd nanostructures is in general better than that of bulk Pd, due to the increased surface-to-volume ratio. This is why low-dimensional Pd nanostructures are so widely investigated notwithstanding the increased difficulty in the fabrication of the requisite nanostructures.

In this review article, we present the H_2_-sensing performance of various sensors employing low-dimensional Pd nanostructures and discuss the finite size effect on the sensor performance, based on results we have obtained up to now. We also highlight issues and possible solutions related to the use of Pd nanostructures. 2D Pd thin films and 1D Pd nanowires are the main subjects of this article with a brief introduction of the use of Pd nanoparticles. The relative strengths and weaknesses of the respective nanostructures are discussed from such various points as performance, manufacturability, and applicability.

## 2. Fundamentals of Electrical Hydrogen Detection

When a Pd is exposed to H_2_, hydrogen molecules are adsorbed onto the Pd surface and dissociated into hydrogen atoms [69]. These hydrogen atoms diffuse until they occupy the interstitial sites of the Pd lattice, causing a certain amount of lattice expansion [70,71]. The diffusion generally takes place through high diffusivity paths such as grain boundaries and dislocations or via a vacancy exchange mechanism [70–73], resulting in the defect density dependence of H_2_ intake. The absorbed hydrogen atoms interact with Pd atoms to form Pd hydrides and increase the frequency of scattering events of charge carriers, which directly leads to the resistance increase of the Pd. In this context, it is expected that the magnitude of resistance increase is proportional to the atomic fraction of absorbed hydrogen atoms to Pd atoms, as expressed by the Sieverts’ law below:

(1) $$\text{Sensitivity}\;\propto \;\frac{\left[H\right]}{\left[\text{Pd}\right]}=\frac{1}{K_{S}}{\left({pH}_{2}\right)}^{1/2}$$

where [H] and [Pd] are the respective concentrations of hydrogen atoms and Pd atoms in the Pd-H system, K_S_ is the Sieverts’ constant, and *p*H_2_ is the H_2_ partial pressure in the environment [26,74,75]. Sieverts’ law refers to the bulk Pd-H system in the isothermal state. According to Equation (1), the resistance change with respect to a reference value, termed sensitivity, is proportional to the relative hydrogen concentration in the solid state, which is correlated with the square root of the hydrogen partial pressure in the gas phase. From this equation, it is inferred that the resistance change on exposure to H_2_ is determined by the H_2_ flux impinging the Pd surface, number of hydrogen accommodation sites, and the hydrogen diffusion rate in the Pd.

Pick *et al*. considered such physical factors to calculate the maximum hydrogen in a niobium (Nb) film with a thin Pd overlayer [76]. They found that the maximum atomic fraction of hydrogen atoms inside the Nb film in equilibrium, *x*_max_, was given as:

(2) $$x_{\text{max}}\;=\;\frac{\nu }{\beta }{\left(\frac{2\Gamma s_{0}}{{\mathrm{KN}}_{S}}\right)}^{1/2}$$

where Γ is the flux of H_2_ molecules hitting the surface of the Pd film, s_0_ is the sticking coefficient of the Pd film, *N_s_* is the number of Nb atoms per unit surface area. *K*, *β*, and *ν* are the Arrhenius type of rate constants for three different fluxes either in vacuum to Nb direction (*ν*) or in Nb to vacuum direction (*K*, *β*). Taking into account that Γ is proportional to H_2_ pressure, Equation (2) is another form of Sieverts’ law that applys to the film system. Cabrera and his colleague derived an empirical relationship between relative resistance change of a Pd film and H_2_ partial pressure with changing the Pd film thickness, which is expressed as [77]:

(3) $$\left(\frac{{\Delta R}_{\text{max}}}{R}\right)\times 100\;=\;1.9{\left({pH}_{2}\right)}^{1/2}$$

Given the previous reports that the resistance increase in a film is proportional to hydrogen concentration absorbed in the film, Equation (3) indicates that Pd thin films also obey the Sieverts law mentioned above. Now that the resistance increase in Pd upon exposure to H_2_ is attributed to the absorbed hydrogen atoms, the concentration of which is strongly correlated with H_2_ partial pressure in the environment, it would be interesting to identify the key process to determine the absorption process.

Kay *et al*. demonstrated that the hydrogen absorption rate of Pd is limited by the diffusion process of hydrogen atoms in the bulk rather than the chemisorption of H_2_ molecules on the surface of the Pd, from an elaborate experiment using a crystalline Pd (110) sheet and a well-designed absorption cell [69]. According to their analyses, a time to half absorption (*t*_1/2_), which is the time for the gaseous H_2_ pressure to reach half of its final value, is given by:

(4) $$t_{1/2}\;=\;-\frac{l^{2}}{\pi^{2}D}\text{ln}\;\left(\frac{\pi^{2}}{16}-\frac{1}{9}{\left(\frac{\pi^{2}}{16}\right)}^{9}\right)$$

where *l* is the sample thickness and D is the hydrogen diffusion coefficient in the Pd. From Equation (4), *t*_1/2_ depends only on the thickness of Pd sample and bulk diffusion coefficient of hydrogen atoms, reflecting that hydrogen absorption in Pd is basically bulk-diffusion-limited process. To validate this hypothesis, they demonstrated that the experimentally determined *t*_1/2_ is independent of the H_2_ pressure in the environment. In addition, they determined the diffusion coefficient and activation energy for the Arrhenius type of hydrogen bulk diffusion to be 2.83 × 10^−3^ cm^2^/s and 5.39 kcal/mol, respectively.

**Figure 1.**
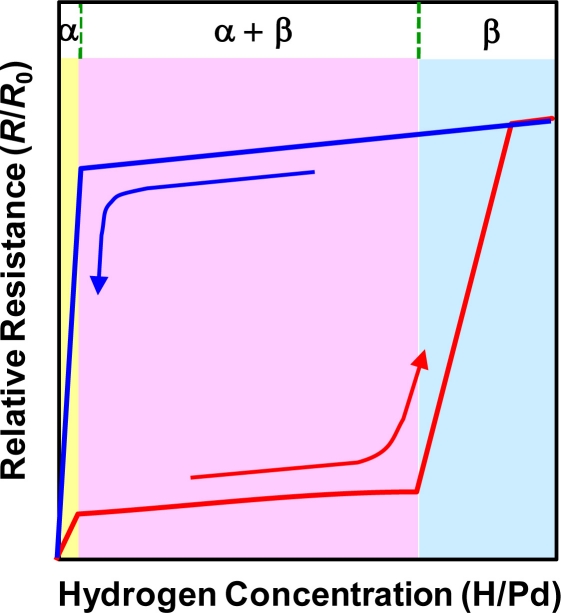
Schematic illustration of relative resistance (*R*/*R*_0_) as a function of relative hydrogen concentration (H/Pd) for absorption-desorption processes. The arrows indicate the directions of absorption and desorption processes.

Although the above description illustrates well the general features of the electrical resistance change of Pd in the presence of H_2_, it is appropriate only for the α phase of the Pd hydride, where a lattice expansion caused by hydrogen filling in the interstitial sites is smaller than 0.13%: *a*_0_ = 0.3890 nm for pure Pd *vs. a*_α, max_ = 0.3895 nm for the upper boundary of the α Pd hydride. As a matter of fact, the Pd hydride, PdH*_x_*, exists in a different phase depending on the atomic fraction of hydrogen (*x*) relative to Pd: α phase when *x* < 0.015, α + β mixed phase when 0.015 ≤ *x* < 0.61, and β phase when *x* > 0.61 at 278 K. Once the concentration of the absorbed hydrogen atoms exceeds the α phase boundary, nuclei of the β phase start to form in the α matrix. The β nuclei grow until the whole original α matrix is transformed to the β phase, where the lattice expansion reaches 3.47%: *a*_0_ = 0.3890 nm *vs. a*_β, min_ = 0.4025 nm for the lower boundary of the β Pd hydride. The hydrogen concentration range for coexistence of the α and β phases depends on temperature and it grows broader as temperature decreases below ∼300 °C, indicating smaller interstitial sites in the α phase and higher density of imperfections in the β phase at lower temperatures. Sakamoto *et al*. carefully investigated a relationship between hydrogen gas pressure (*p*H_2_), hydrogen concentration in Pd (H/Pd), and relative resistance in the pressure (*R*/*R*_0_) at a specific temperature, using a gas phase method [70]. In contrast with the previous monotonic increase in resistance with hydrogen concentration in Pd, as expressed by Sieverts’ law, they obtained three characteristic regions with different rates of resistance increase. They correspond to the α phase, α + β mixed phase, and β phase, respectively, as schematically shown in Figure 1.

In Figure 1, the resistance traces are different for absorption and desorption processes, which is a characteristic hysteretic resistance behavior of Pd upon cyclic exposure to H_2_. The abrupt increase in resistance in the β phase region is attributed to the plastic deformation caused mainly by dislocation formation and pile-up. As mentioned above, the appearance of the β phase is dependent on temperature, the steep resistance increase in the β phase and the following hysteresis during desorption process can be alleviated at elevated temperatures. Provided that room-temperature operation is a basic requirement, however, the hysteretic resistance behavior and plastic deformation in the high H/Pd region may be potential serious issues of Pd-based sensors. These issues likely become more serious in low-dimensional Pd structures, in particular, Pd thin films, requiring close investigation on the issues followed by a search for the possible solutions.

## 3. Two-Dimensional Pd Thin Films

Pd thin films are a basic form of low-dimensional Pd nanostructure, which are easily adaptable to practical hydrogen sensor applications. In these structures, the thickness of the films is usually restricted to less than several hundreds of nm, while the width is flexibly varied to fit into a allowed dimension in the range of several mm to several cm. The very low aspect ratio of film thickness to width generally facilitates hydrogen absorption and desorption processes, and makes the Pd film/substrate interface profoundly important in the cyclic hydrogen sensing. Here, we present the hydrogen-sensing properties of Pd thin films, discuss critical issues related to these film structures, and suggest the possible solutions to tackle the issues.

### 3.1. Pure Pd Thin Films

**Figure 2.**
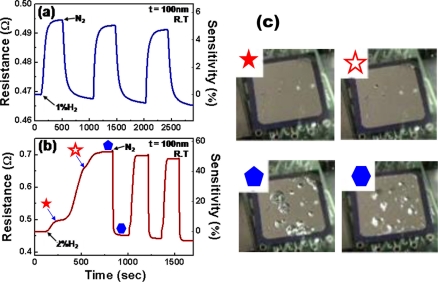
Electrical resistances and the corresponding sensitivities of a 100 nm-thick Pd thin film in response to **(a)** 1% H_2_ and **(b)** 2% H_2_ at room temperature. **(c)** Film morphologies at the steps indicated by the respective symbols in (b). (a) and (b) Data reproduced from Lee *et al*. [77] and (c) reproduced from Kim *et al*. [78].

The hydrogen-sensing properties of Pd thin films on thermally oxidized Si substrates were investigated at room temperature, using nitrogen carrier gas (N_2_). Figure 2 shows the two representative real-time electrical responses of a 100 nm-thick Pd film for 1 and 2% H_2_. Hereafter, the sensitivity is defined as:

(5) $$\text{Sensitivity}\;(\%)=\frac{R_{H}-R_{N}}{R_{N}}\times 100$$

where *R*_H_ and *R*_N_ are the resistances in the presence of H_2_ and N_2_ gases, respectively. The Pd film behaves like a reversible hydrogen sensor with sensitivities proportional to H_2_ concentration, reaching 52% at 2% H_2_. One interesting observation is that unlike the response to 1% H_2_, which shows monotonic resistance increase with the absorption of H_2_ (Figure 2(a)), several intermediate stages with reduced rates of resistance increase appear around the inflection points in the first cycle of response to 2% H_2_ as indicated by star marks in Figure 2(b). These intermediate stages signal the occurrence of new phases, *i.e.*, α + β mixed phase (★) and β phase (✰). Similar to the case in Figure 1, the β phase nucleates in the α matrix if the amount of hydrogen incorporation into the interstitial sites of Pd exceeds the solid solubility limit of hydrogen, and grows with the further absorption of H_2_. In this α + β mixed phase, the resistance increase is more likely to be governed by the relative volume fraction and distribution of β islands rather than total atomic fraction of hydrogen. Once the phase transition to the β phase is completed, defects such as vacancies and dislocations are induced into the matrix both to maintain the NaCl structure of the PdH*_x_* and to release the strain produced by gradual volume expansion by absorbed hydrogen atoms. A further increase in hydrogen concentration causes severe structural deformations (see the image ( 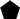) in Figure 2(c)) and the deformations are only partially recovered even after H_2_ supply is disconnected (see the image ( 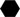) in Figure 2(c)).

**Figure 3.**
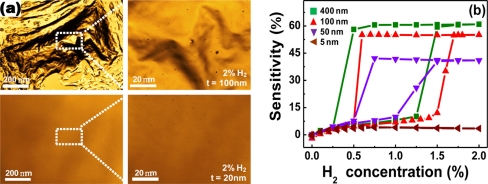
**(a)** Confocal laser scanning microscopy images of the Pd thin films with the thicknesses of 100 and 20 nm, respectively, after exposure to 2% H_2_. **(b)** Sensitivity *vs*. H_2_ concentration curves for Pd films with different thicknesses of 5 to 400 nm undergoing cyclic H_2_ absorption and desorption processes. Data reproduced from Lee *et al*. [77].

Serious structural deformations can also be observed by confocal laser scanning microscopy. Figure 3(a) shows the microscopic surface scan images of Pd films in exposure to 2% H_2_, using this technique. The severe film delamination is observed for the Pd film with a thickness of 100 nm, in accordance with the results above. Interestingly, the degree of structural deformations depends on the thickness of Pd film and only small pits are observed on the 20 nm-thick Pd film.

Because the structural deformations are only partially recovered after stopping the H_2_ flow, they give rise to a hysteresis in resistance *vs*. H_2_ concentration curves for a cycle of H_2_ absorption and desorption processes, as shown in Figure 3(b). The magnitude of the hysteretic behavior, however, decreases with decreasing Pd film thickness, which is well-matched to the tendency observed in thickness-dependent surface deformations. From Figure 3(b), the maximal Pd film thickness, where no hysteresis is observed, is found to be 5 nm, at which the sensitivity is only 4%. It is also difficult to precisely control the thickness as thin as this.

### 3.2. Pd-Ni Alloy Thin Films

**Figure 4.**
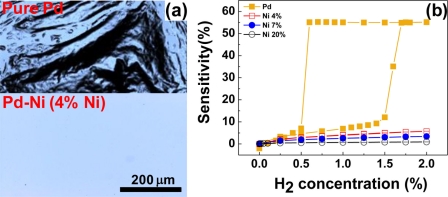
**(a)** Confocal laser scanning microscopy images of a pure Pd and a Pd-Ni alloy (4% Ni) films after exposure to 2% H_2_. **(b)** Sensitivities of a pure Pd film and Pd-Ni alloy films with varying Ni content as a function of H_2_ concentration at room temperature. Data reproduced from Lee *et al*. [82].

**Figure 5.**
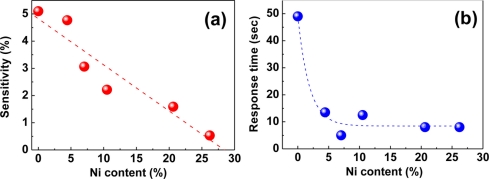
**(a)** Sensitivity and **(b)** response time of Pd-Ni alloy films as a functions of Ni content in the presence of 1% H_2_ at room temperature. Data reproduced from Lee *et al*. [82].

The severe structural deformations and the consequent hysteretic resistance behaviors of pure Pd films should find solutions since they risk the reliability and accuracy of the Pd film-based hydrogen sensors. Various Pd alloys such as Pd-Mg, Pd-Au, Pd-Ag, and Pd-Ni have been explored to enhance the structural stability of the Pd-based thin films of the Pd-based thin films [40,79–82]. Among those, Pd-Au and Pd-Ag alloys have been more studied due to their advantages such as crystal structures similar to that of Pd, higher hydrogen solubilities than pure Pd, and good capabilities to suppress structural deformations. However, they have problematic issues too. For instance, the Au in the Pd-Au alloy easily segregates on the surface owing to its high mobility [83], causing a reliability problem. For the Pd-Ag alloy, good sensor performance, particularly, high hydrogen permeability is observed only in high Ag concentration more than 20 wt% and the hydrogen dissociation capability of the alloy becomes worse than pure Pd in this concentration range [84]. Moreover, both alloys are expensive, adding more cost to sensors incorporating those materials. Comparing Pd-Ni alloy to those alloys, Ni can be introduced to Pd matrix in less amount for suppressing structural deformations. Ni is less likely to segregate in the alloy, is inexpensive, and helps to shorten response times. The attributes of the Pd-Ni alloy like good durability, fast response, and consistency of crystal structure with Pd make it attractive material for hydrogen sensing. According to our investigation, the addition of a small amount of Ni to Pd effectively suppresses the structural deformations, as demonstrated in Figure 4(a). A Pd-Ni alloy containing 4% Ni shows no conceivable surface defects, leading to hysteresis-free resistance change in response to H_2_ concentration. From Figure 4(b), it is found that the resistances of Pd-Ni alloy films containing more than 4% of Ni increase almost linearly with H_2_ concentration without a hysteresis, while a pure Pd film shows a sizable hysteretic behavior in its resistance change. Although these results are desirable for sensor operations, the sensitivities of the Pd-Ni alloys are smaller than that of pure Pd. The small sensitivities and linear relationships between the sensitivity and H_2_ concentration of Pd-Ni alloys are ascribed to a reduced H_2_ in-take and the hindered β phase formation due to the smaller lattice constants and interstitial volumes and stronger Pd-Ni bonds with respect to pure Pd (see Figure 5(a) below for the dependence of sensitivity on Ni content).

In contrast with the reduced sensitivities, the Pd-Ni alloy films respond to H_2_ much faster than a pure Pd film, as seen from Figure 5(b).

The response time drastically drops by a factor of ∼5 from the value of a pure Pd when 4% of Ni is added to Pd, and then it remains almost unchanged with further Ni addition. Here, the response time is defined as the time required to reach 36.8% (=e^−1^) of the total resistance change at a given H_2_ concentration. Owing to the difference in lattice constants of Pd (3.891 Å) and Ni (3.524 Å), imperfections such as grain boundaries and dislocations are likely to be formed to compensate for the lattice strain generated by the addition of a small amount of Ni to the Pd matrix. These imperfections may function as a preferential diffusion path for the absorbed hydrogen atoms, leading to the shorter response time for Pd-Ni alloys. The steady response time appearing beyond ∼7% Ni stems possibly from the deterioration of crystal quality at high Ni contents rather than the further creation of the line or plane defects. This fast response of the Pd-Ni alloys as well as the sensitivity proportional to H_2_ concentration makes these alloys a class of good materials for fast, reliable, and scalable H_2_ sensing.

### 3.3. Pd Thin Films on a Ti Buffer

**Figure 6.**
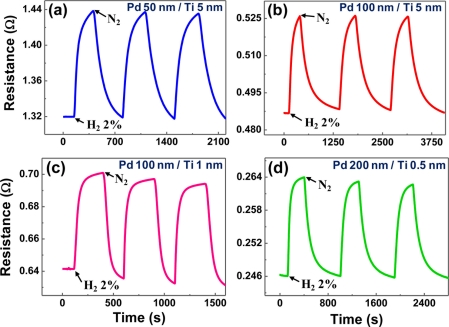
The real-time electrical responses of Pd films on a Ti buffer layer with different combinations of Pd and Ti layer thicknesses: **(a)** 50(Pd)/5(Ti) nm, **(b)** 100/5 nm, **(c)** 100/1 nm, and **(d)** 200/0.5 nm. Measurements were performed at room temperature, using 2% H_2_. Data reproduced from Kim *et al*. [78].

Although the Pd-Ni alloys with a Ni content less than 10% achieved a very fast and hysteresis-free response, the introduction of Ni not only complicates the film preparation, but is also incompatible with the conventional semiconductor integration process. Moreover, the Pd-Ni alloys suffer from a small sensitivity even to high H_2_ concentration (∼5.5% at 2% H_2_) and a resistivity increased from the value of Pd even at low Ni content (6 to 7 times at 7% Ni). The structural deformation and hysteretic resistance problems of pure Pd films can be more easily treated using a thin buffer layer. Titanium (Ti) is a typical adhesion promoter for thin films, that is widely used in the present semiconductor industry. We closely examined the effects of a thin Ti buffer layer on the structural and electrical properties of Pd thin films. The film delamination that was a severe problem for pure Pd films was not observed. Real-time electrical responses at room temperature are shown in Figure 6 for Pd/Ti film stacks with different combinations of respective layer thicknesses. All curves exhibit monotonic resistance increase without any intermediate stages, reflecting the absence of structural deformations. Comparing the 100 nm-thick Pd films without (described above) and with Ti buffer layers (5 and 1 nm), the sensitivities (8% and 9.2%) of Ti-buffer Pd films are smaller than that (∼52%) of a pure Pd film.

This indicates that the Ti-mediated improvement in adhesion of Pd film to the substrate strengthens so-called ‘clamping effect’ and restricts global volume expansion of the Pd film [85], lowering the level of H_2_ in-take and suppressing the appearance of the β hydride. The small increase (only 1.2%) of sensitivity for the thinner Ti buffer reflects that the reinforcement of the clamping effect occurs primarily in the vicinity of Pd film/Ti buffer interface, as also supported by the inflectionless response for even thinner Ti (0.5 nm) in Figure 6(d). Considering the response time, the Ti-buffered Pd films apparently respond to H_2_ faster (20–30 s for Figure 6(a) through (d)) than does a Pd film with a comparable thickness (∼50 s for 100 nm-thick Pd film). The decreased response time of the Ti-buffered Pd films is attributed to the reduced distance for hydrogen diffusion due to the reduced free volume by the reinforced clamping effect in these film stacks.

**Figure 7.**
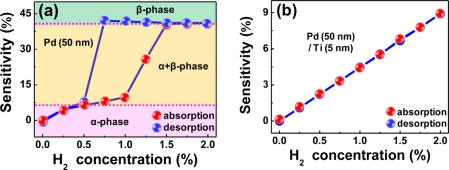
Sensitivity changes in **(a)** a pure Pd film and **(b)** a Ti-buffered Pd film as a function of H_2_ concentration in H_2_ absorption and desorption processes. The Pd film and Ti buffer layer thicknesses were 50 and 5 nm. Data reproduced from Kim *et al*. [78].

As explained above, the structural deformations and hysteretic response behaviors of pure Pd films are associated with phase transitions in them, as represented by Figure 7(a). The β phase grows as H_2_ concentration increases and the tensile stress produced by volume expansion gradually imposes onto the film/substrate interface. Once this stress exceeds the Pd-substrate bond strength, the Pd film starts to deform until it is delaminated from the substrate, resulting in an abrupt resistance increase. Because these structural deformations are mostly irreversible, there appears a lag in resistance recovery to the α phase value in the desorption process.

The complete phase transition from the α to β phase occurs at ∼1.5% H_2_. In contrast, the H_2_ in-take and β phase formation are restricted in the Ti-buffered Pd films due to the enhanced clamping effect, leading to a linear relationship between the sensitivity and H_2_ concentration in both H_2_ absorption and desorption processes without a hysteresis, as shown in Figure 7(b). Furthermore, the sensitivity of this Ti-buffered Pd film is larger than that of Pd-Ni alloy films by a factor of 1.6 to 1.7 at a given H_2_ concentration. The reasonable sensitivity and fast response of the Ti-buffered Pd films without structural deformations and hysteresis, along with a simple fabrication process, allow the film stacks to be considered for practical applications in hydrogen sensors.

### 3.4. Nanoporous Pd Thin Films

Despite the many strong points the conventional dense Pd films have as hydrogen sensors, they are generally difficult to use for measuring H_2_ concentration lower than 500 ppm with precision and their response time still needs to be improved. To address these issues, Pd thin films with nanopores have been investigated using anodic aluminum oxide (AAO) templates. The AAO-supported Pd thin films are expected to show a lower H_2_ detection limit and faster response than the dense Pd films due to the greatly increased surface area. In addition, the film delamination problem may be alleviated in the nanoporous structures owing to the potentially enhanced adhesion provided by the heavy up and down substrate structure. Ding *et al*. fabricated AAO-supported nanoporous Pd thin films and compared their response characteristics with those of dense Pd films [86]. Notably, the thin porous Pd film could clearly detect H_2_ concentrations as low as 250 ppm. Comparing the responses of a dense Pd and a porous Pd films with an identical thickness of 45 nm, the resistance change in the porous film was faster and sharper than that of the dense film. The faster and sharper response and lower detection limit of the thin nanoporous Pd film probably originate from the dual contributions of a very thin thickness effect and an enlarged surface effect.

## 4. One-Dimensional Pd Nanowires

Through the study of nanoporous Pd films described above, it was demonstrated that the H_2_ detection limit and response time could be reduced in nanoporous structures due to their increased surface area. If it is the real case, Pd nanowires would be ideal structures for fast detection of low H_2_ concentrations. However, the H_2_-sensing properties of the Pd nanowires should be considered along with methods for their fabrication, because nanowires are in general harder to fabricate compared to thin films and their properties depend on the final structures. Here, we present the H_2_-sensing properties of lithographically patterned Pd nanowires, bottom-up grown Pd nanowires, ion-milled Pd nanowires, and grain-structured Pd nanowires. Finite size effects in the Pd nanowires are also discussed.

### 4.1. Lithographically Patterned Pd Nanowires

**Figure 8.**
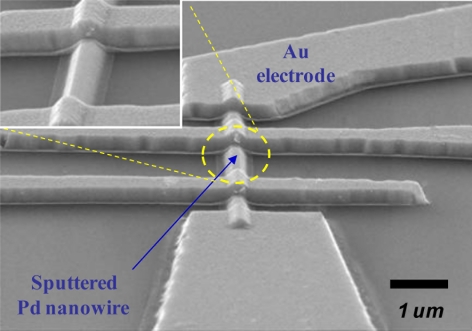
SEM image of a lithographically patterned Pd nanowire with *t* = 100 nm, *w* = 300 nm, and *l* = 10 μm. Four Ti/Au inner electrodes were patterned on the Pd nanowire. Data reproduced from Jeon *et al*. [87].

Lithographically patterned Pd nanowires can be regarded as intermediate structures between Pd thin films and bottom-up grown Pd nanowires. To fabricate these nanostructures, a combination of E-beam lithography and a lift-off process was used. Pd thin films with varying thickness *t* = 20–400 nm were sputter-deposited on thermally oxidized Si substrates. The patterned Pd nanowires had an identical width *w* = 300 nm and length *l* = 10 μm. Later, different combinations of a lift-off process and either photolithography or E-beam lithography were used to pattern relatively larger Ti/Au outer electrodes and micron-scaled Ti/Au inner electrodes, respectively, on the Pd nanowires. Figure 8 shows a SEM image of a lithographically patterned Pd nanowire (*t* = 100 nm) with four inner electrodes.

**Figure 9.**
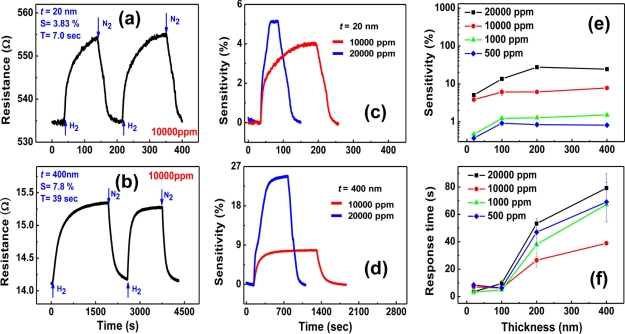
The real-time electrical responses of lithographically patterned Pd nanowires with **(a)** *t* = 20 nm and **(b)** *t* = 400 nm to 10000 ppm of H_2_ at room temperature. Sensitivities upon exposure to 10,000 and 20,000 ppm of H_2_ for the Pd nanowires with **(c)** *t* = 20 nm and **(d)** *t* = 400 nm. **(e)** Sensitivity and **(f)** response time as a function of the thickness of lithographically patterned Pd nanowires in the H_2_ concentration range of 500 to 20,000 ppm. Data reproduced from Jeon *et al*. [87].

H_2_-sensing properties of the lithographically patterned Pd nanowires were investigated at room temperature as a function of H_2_ concentration and nanowire thickness. Typical results are shown in Figure 9. Figures 9(a,b) exhibit electrical responses of two Pd nanowires with *t* = 20 and 400 nm, respectively, at the same H_2_ concentration of 10,000 ppm. The thinner nanowire (*t* = 20 nm) shows a lower sensitivity, but a much faster response time compared to the thicker one (*t* = 400 nm). This difference is attributed to the stronger clamping effect in the thinner nanowire, as explained in the previous section of Pd thin films. Because the clamping effect originates from the nanowire/substrate interface, it is stronger at positions closer to the interface. The stronger clamping effect in the thinner nanowire suppresses the free volume expansion of the nanowire to limit the hydrogen absorption, leading to a lower sensitivity. The shorter response time is obtained by the reduction of hydrogen diffusion distance in the thinner nanowire.

The relative intensity of the clamping effect can be deduced from the difference in sensitivities to 10,000 and 20,000 ppm H_2_, as shown in Figures 9(c,d). The ratio of sensitivities to respective 10,000 and 20,000 ppm H_2_ for the thinner nanowire (*t =* 20 nm) is only 1.3, in contrast with 3 for the thicker nanowire (*t =* 400 nm). This indicates that the thinner nanowire absorbs less hydrogen atoms than the thicker one does at the same concentration gradient since the resistance increase upon H_2_ exposure is basically caused by the enhanced carrier scattering by the absorbed hydrogen atoms, justifying the stronger clamping effect in the thinner Pd nanowire.

Figures 9(e,f) show comprehensive trends of sensitivity and response time as functions of nanowire thickness and H_2_ concentration. The sensitivity is found to increase with increasing H_2_ concentration for all thicknesses, as shown in Figure 9(e). However, it is almost saturated beyond a certain thickness, particularly 100 nm at H_2_ concentrations lower than 10,000 ppm. This may be because the weakening of the clamping effect is counterbalanced by the reduced surface-to-volume ratio in this thickness range. The larger saturation thickness (200 nm) at a higher H_2_ concentration (20,000 ppm) reflects that the relaxation of the clamping effect is more effective than the reduction of the surface-to-volume ratio at a high H_2_ partial pressure, probably supported by the slight structural deformation. These sensitivity trends in lithographically patterned Pd nanowires are different from the observations in Pd thin films, where the sensitivity increases with increasing the film thickness in the range of 5 to 400 nm and hysteretic resistance behaviors are observed in all films thicker than 20 nm due to the α to β phase transitions. As a matter of fact, the lithographically patterned Pd nanowires with thicknesses smaller than 100 nm showed an almost linear relationship between the sensitivity and H_2_ concentration. As a consequence, it was demonstrated that a thin Pd nanowire could detect H_2_ concentrations as low as 20 ppm.

On the contrary, the response time remains almost constant below 100 nm whereas it gradually decreases with decreasing thickness down to this value. The general decrease of response time with decreasing the thickness is due to the reduced hydrogen diffusion distance mentioned above. The steady response time along with the sensitivity trend in the thickness range smaller than 100 nm suggests that the clamping effect dominates the hydrogen absorption dynamics in this range. The shortest response time obtained from the lithographically patterned Pd nanowires was ∼3 s at 1,000 ppm of H_2_ for one with *t* = 20 nm, when the response time is defined as the time required to reach 36.8% (=e^−1^) of the total resistance change as before. This is much smaller than that of Pd thin films.

### 4.2. Bottom-Up Grown Pd Nanowires

**Figure 10.**
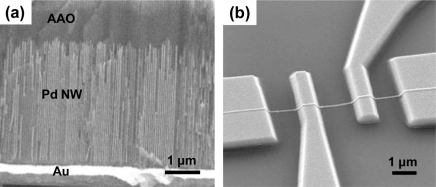
**(a)** Pd nanowire arrays grown by electrodeposition into nanochannels of AAO templates. **(b)** A representative four terminal device on an individual Pd nanowire. Data reproduced from Jeon *et al*. [88].

It was found that Pd nanowires fabricated by lithography techniques could be good hydrogen sensors with fast response and low detection limit. However, they possibly face the risk of structural deformations at high H_2_ concentrations because the nanowire body sticks to the substrate. This problem would be eliminated using bottom-up grown Pd nanowires, which have no direct bonds with the substrate. It would be possible to investigate more intrinsic finite size effects of H_2_-sensing properties using these Pd nanowires because the clamping effect could be ruled out in these isolated nanowire structures. As an example of this bottom-up approach, Pd nanowires were grown by electrodeposition into nanochannels of AAO templates from an aqueous solution containing 0.034 mol of PdCl_2_, using an Au cathode layer (see Figure 10(a)).

Pd nanowires were rinsed and immersed in isopropyl alcohol (IPA) after chemically removing the AAO templates. The Pd nanowires were dispersed onto a thermally oxidized Si substrate with patterned outer electrodes on it by a drop-casting method. A combination of E-beam lithography and a lift-off process was used to pattern inner Au electrodes on an individual Pd nanowire. A representative four terminal device on the individual Pd nanowire is shown in Figure 10(b).

**Figure 11.**
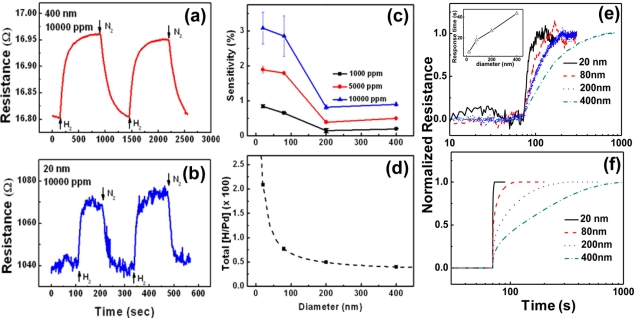
The real-time electrical responses of bottom-up grown Pd nanowires with **(a)** *d* = 400 nm and **(b)** *d* = 20 nm to 10,000 ppm of H_2_ at room temperature. **(c)** Sensitivity as a function of nanowire diameter in the H_2_ concentration range of 1,000 to 10,000 ppm. **(d)** The calculated atomic fraction of hydrogen to Pd as a function of nanowire diameter in the presence of 10,000 ppm H_2_. **(e)** The experimental and **(f)** calculated results of normalized resistances at 10,000 ppm H_2_ for Pd nanowires with varying diameters. The inset of (e) shows a change in the response time with nanowire diameter. Data reproduced from Jeon *et al*. [88].

H_2_-sensing properties of the bottom-up grown Pd nanowires were investigated at room temperature as functions of H_2_ concentration and nanowire diameter in a similar manner for the previous lithographically patterned Pd nanowires. Figures 11(a,b) show representative electrical responses of two Pd nanowires with *d* = 20 and 400 nm, respectively, at the same H_2_ concentration of 10,000 ppm. On the contrary to lithographically patterned nanowires with the same thicknesses, the thinner nanowire (*d* = 20 nm) exhibits a larger sensitivity than that of the thicker one (*d* = 400 nm). This trend can be confirmed more clearly from the nanowire diameter dependence of the sensitivity shown in Figure 11(c). It is found from this figure that the sensitivity of the Pd nanowire increases with decreasing the nanowire diameter in a range of 0–200 nm for H_2_ concentrations tested. Since the sensitivity is proportional to the relative concentration of hydrogen atoms in Pd matrix (∝ [H]/[Pd]), this atomic ratio was calculated by the following equation:

(6) $$\begin{array}{c} {}[H]/[\text{Pd}]=\frac{2\pi \frac{d}{2}l(X_{1}(t)X_{1s}+X_{2}(t)X_{2s})+\pi {\left(\frac{d}{2}\right)}^{2}lY(t)Y_{b}}{2(2\pi \frac{d}{2}{\mathrm{lN}}_{s})+\pi {\left(\frac{d}{2}\right)}^{2}{\mathrm{lN}}_{b}}\\ =\frac{2[X_{1}(t)X_{1s}+X_{2}(t)X_{2s}]+\frac{d}{2}Y(t)Y_{b}}{{4N}_{s}+\frac{d}{2}N_{b}} \end{array}$$

where *l* is the nanowire length, *X*_1_(*t*) and *X*_2_(*t*) represent the occupation probability of hydrogen atoms in the respective sites at the surface (*X*_1s_ = 9.4 × 10^14^ sites/cm^2^) and subsurface (*X*_2s_ = 4.7 × 10^14^ sites/cm^2^), and *Y*_b_ represents the site density of the bulk part (*Y*_b_ = 4.7 × 10^20^ sites/cm^3^). Similarly, *N*_s_ (1.69 × 10^15^ atoms/cm^2^) and *N*_b_ (6.81 × 10^22^ atoms/cm^2^) are atomic concentrations of Pd at the surface and in the bulk, respectively. *Y*(*t*) denotes the normalized hydrogen concentration of the bulk part, which is governed by the Fickian diffusion process as follows:

(7) $$\frac{\mathrm{dY}(r,t)}{\mathrm{dt}}=\text{exp}\left(-\frac{E_{D}^{*}}{\mathrm{RT}}\right)\frac{1}{r}\frac{d}{\mathrm{dr}}\left(r\;\frac{\mathrm{dY}(r,t)}{\mathrm{dr}}\right)$$

where *r* is the radial distance from the nanowire center and *E*_D_^*^ is the activation energy of the diffusion process. The calculation result is given in Figure 11(d). The relative fraction of hydrogen atoms to Pd atoms rapidly increases as the nanowire diameter decreases, which is similar to the experimental results shown in Figure 11(c). This is because the relative hydrogen-accommodating site density to Pd atom density at the surface ((*X*_1s_ + *X*_2s_)/2*N*_s_) is much larger than that in the bulk and the occupation probability of hydrogen atoms at the surface ((*X*_1_ + *X*_2_)/2) is also higher than in the bulk. The steady (or slow) change in the sensitivity (or in [H]/[Pd]) in nanowire diameters larger than 200 nm may result from the rapidly decreasing contribution of the surface layers to hydrogen diffusion due to the drastic reduction of the surface volume fraction with increasing the diameter. Compared with the previous lithographically patterned nanowires, the sensitivity (∼3%) of the bottom-up grown Pd nanowires is found to be slightly smaller, reflecting that H_2_ absorption occurs dominantly over the surface region in these isolated nanowires. Figures 11(e,f) show the experimental and calculation results of the nanowire diameter dependence of response time. From both figures, it is seen that the response time gradually decreases as the diameter decreases. This is because hydrogen diffusion is accelerated at a small diffusion distance (diameter) as also indicated by Equation (7). The shortest response time measured in these bottom-up grown Pd nanowires was ∼6 s, which is similar to that in lithographically patterned nanowires.

### 4.3. Ion-Milled Pd Nanowires

**Figure 12.**
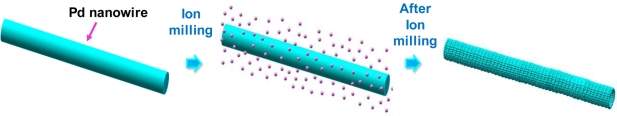
Schematic illustration of the rough surface of Pd nanowires induced by ion-milling.

From the study of the bottom-up grown Pd nanowires, it was found that the sensitivity increases while the response time decreases as the nanowire diameter shrinks. This was mainly ascribed to the higher density of hydrogen-accommodating sites at the surface compared to the bulk. If this is the case, it would be interesting if we investigate the effects of Pd nanowire surface roughness on the H_2_-sensing performance because the rough nanowire would provide the larger surface area than the smooth one at the same average diameter. To check this effect, Lee *et al.* [89] intentionally induced a rough surface on electrodeposition-grown Pd nanowires using ion-milling (beam energy: 500 eV, beam current density: 5 μA/cm^2^, milling time: 10 min), as shown schematically in Figure 12. The surface of the Pd nanowires, which was initially smooth, appeared to be rough after ion-milling, presumably due to different etch rates depending on crystal planes. They measured electrical responses on individual Pd nanowires before and after ion-milling. Similar device structures to that shown in Figure 10(b) were used for the response measurements. Surprisingly, the resistance of the ion-milled nanowire increased much faster than the as-grown nanowire. When defining the response time as the time required to reach 90% of the total resistance change, the response time of the ion-milled Pd nanowire was found to be approximately 20 times shorter than that of the as-grown one (25 and 500 s, respectively). This is attributed to the enhanced surface-to-volume ratio and the consequent increase of hydrogen-accommodating sites of the ion-milled Pd nanowire. Interestingly, sensitivity seems almost unchanged even through the ion-milling. It may be because the sensitivity depends on the total nanowire volume (average diameter) rather than the surface area.

### 4.4. Grain-Structured Pd Nanowires

The Pd nanowires grown by electrodeposition into AAO templates described above were structurally homogeneous, resulting in the resistance increase upon H_2_ exposure. However, many electrodeposited Pd nanowires can have different morphologies depending on the growth conditions. Hu *et al*. [90] and Yang *et al*. [91] have demonstrated that the electrodeposited Pd nanowires with different morphologies led to sharp contrasts in their respective response behaviors. Hu *et al*. grew Pd nanowires on polymethylmethacrylate (PMMA) channels between two Au electrodes by electrophoresis, by varying growth currents [90]. They obtained three different kinds of nanowire structures depending on the nanowire diameter: plain structure at *d* = 85 nm, grain structure in the range of *d* = 100–150 nm, and hairy structure at *d* = ∼100 nm. Interestingly, Pd nanowires with grain structure exhibited the inverse-type of resistance behavior (Δ*R*/*R*_0_ < 0), while normal resistance behaviors (Δ*R*/*R*_0_ > 0) were observed in nanowires with plain structure. The grain-structured nanowires are composed of Pd nanoparticles either in close contact or connected by narrow necks. When these Pd nanowires are exposed to H_2_, the Pd nanoparticles expand to reduce the length of the neck and the neck itself expands at the same time. These expansions result in an increase in contact area between neighboring nanoparticles, leading to the significant decrease in nanowire resistance. The expanded Pd nanoparticles and necks are back to the original states as soon as H_2_ flow stops. The grain-structured Pd nanowires could detect H_2_ concentrations as low as 2–5 ppm. Pd nanowires with hairy structure were subdivided into two different internal structures, which showed normal and inverse resistance behaviors, respectively. The Pd nanowire arrays with grain structures prepared by dielectrophoresis on patterned Si substrates, which was performed by La Ferrara *et al*. [7], showed a high sensitivity up to 140% at 4% H_2_. Furthermore, thinner nanowires were found to produce a higher sensitivity and a shorter response time, which is in good agreement with the trend observed in more uniform, isolated Pd nanowires stated above.

## 5. Pd Nanoparticles

Despite the interesting H_2_-sensing properties (fast response, reasonable sensitivity, and good reproducibility) of the bottom-up grown homogeneous Pd nanowires, precision diameter control and device fabrication on the nanowires are challenging. Recollecting that the high performance of the nanowires is ascribed to the high surface-to-volume ratio of the structures and high hydrogen-accommodating site density at the surface, Pd nanopartices are expected to show better performance although they have a difficulty in fabrication. Because using a single Pd nanopartice as a sensor medium is extremely difficult, continuous films of Pd nanoparticles or other structures incorporating them have been exploited. Here, we briefly present the examples of using Pd nanoparticles as H_2_ sensors: continuous films consisting of Pd nanoparticles and Pd nanoparticles grafted onto carbon nanotubes (CNTs).

### 5.1. Continuous Films of Pd Nanoparticles

**Figure 13.**
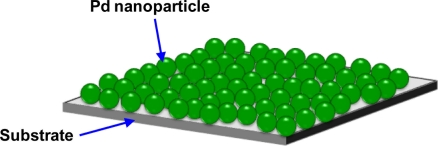
Schematic representation of a continuous film of Pd nanoparticles.

Joshi *et al*. electrochemically deposited Pd nanoparticles on Si substrates by electrodeposition from a solution of Pd sulfate and sulfuric acid [92]. The deposited nanoparticles were dense enough to form a continuous film, as shown schematically in Figure 13.

For sensing performance comparison, sputtered Pd thin films were also prepared in parallel. The average grain sizes of Pd nanoparticle films and sputtered films were varied by either changing deposition time or deposition pressure. The Pd nanoparticle film showed the normal resistance behavior with a resistance increase upon exposure to H_2_, confirming that the Pd nanoparticles were electrically connected. Referring to Equation (5) for the estimation of sensitivity, the sensitivity was approximately 100% at 1% H_2_, which was about 70% higher than that of the Pd thin film with the same 150 nm thickness at the same H_2_ concentration (see Figure 3(b)). This indicates that the continuous film of Pd nanoparticles can detect H_2_ with larger signals as compared to the normal Pd thin film. The systematic grain-size-dependent comparison study of the sensitivity and response time between the film of Pd nanoparticles and sputtered Pd thin film was carried out. The sensitivity of a Pd nanoparticle film was higher than that of a sputtered Pd thin film at all H_2_ concentrations and grain sizes, while the response time was shorter in the nanoparticle film than in the sputtered film. Furthermore, for both the nanoparticle film and sputtered film, the sensitivity increased and response time decreased as the average grain size decreases at given H_2_ concentrations. These results reflect that the enlarged surface-to-volume ratio of the Pd nanoparticles and the higher grain boundary density at a film with smaller grains cooperatively contribute to a higher amount of H_2_ in-take and a faster hydrogen diffusion. The hydrogen diffusion is also facilitated by the higher density of hydrogen-accommodating sites at the surface of smaller nanoparticles and grains.

### 5.2. Pd Nanoparticles Grafted onto CNTs

**Figure 14.**
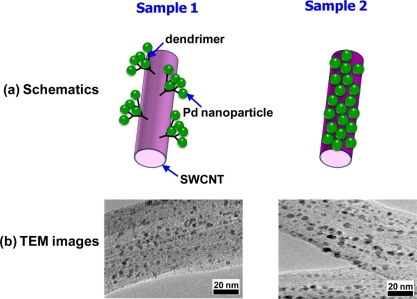
**(a)** Schematic pictures and **(b)** transmission electron microscopy (TEM) images of two types of Pd nanoparticle samples grafted on SWCNTs.

Although the continuous film of Pd nanoparticles demonstrated the improved H_2_-sensing performance of the nanoparticle systems, it was an intermediate structure between the dense Pd film and a cluster of authentic Pd nanoparticles, consisting of rather large nanoparticles (>14 nm). Moreover, the H_2_-sensing mechanism was almost the same as for the normal resistance-based H_2_ sensors. Ju *et al*. fabricated a different type of H_2_ sensor, consisting of single-walled CNTs (SWCNTs) as building blocks, truly small Pd nanoparticles (2–3 nm) as H_2_ absorbers, and dendrimers as grafting mediators of the Pd nanoparticles to SWCNTs [93]. Despite the many advantages of CNTs such as hollow cores, small size, and large surface area, they hardly interact with H_2_ and need to be functionalized to be used as H_2_ sensors. Ju *et al*. first pretreated the SWCNTs to form terminal amine groups on their surfaces and then chemically grew polyamidoamine dendrimers on the surfaces of the pretreated SWCNTs. Subsequently, Pd nanoparticles were grafted onto the dendrimer-modified SWCNTs in aqueous solution of PdCl_4_^2−^ and NaBH_4_ (see sample 1 in Figure 14). This sample further underwent a pyrolysis (200 °C, 12 h) to remove the dendrimers (see sample 2 in Figure 14).

A control sample, where Pd nanoparticles were directly grafted on SWCNTs without dendrimers, were also prepared and Pd nanoparticles appeared to be scarcely formed on its surface. On the other hand, both samples 1 and 2 showed high densities of Pd nanoparticles, confirming the efficacy of the SWCNT modification and nanoparticle-grafting processes. It was believed that the larger nanoparticle size for sample 2 is due to the agglomeration of adjacent nanoparticles during annealing at the elevated temperature (200 °C). Both samples showed normal response behaviors, in which resistance increased upon exposure to H_2_. Comparing the two samples, sample 2 exhibited a higher sensitivity (25%) and a slightly longer response time (7 s) than those of sample 1 to the same H_2_ concentration (10,000 ppm). Because both samples behaved like a normal resistance-based H_2_ sensor, the possibility of engagement of break junctions between Pd nanoparticles should be ruled out. Instead, interactions between the Pd nanoparticles and SWCNTs are the most likely mechanisms to generate such output signals. In this respect, the two samples see different environments and respond differently to H_2_, mainly depending on the presence or absence of dendrimer layer. It is difficult to transfer absorbed electrons from Pd nanoparticles to SWCNTs due to the dielectric layer in-between them (sample 1). Thus, the dipole moment formation mediated by dendrimers is more likely to be a main player in sample 1. Once dipole moments are formed across the dendrimer layer in response to H_2_ absorption, the mobility of majority carriers in SWCNTs becomes lower, leading to an increase in resistance. The response time is short because the dipole moments are formed quickly and the sensitivity is rather small because the influence of this phenomenon is not strong. By contrast, the direct electron transfer is possible in sample 2 since Pd nanoparticles are in contact with SWCNTs. Hence, the absorbed hydrogen atoms lower the work function of the Pd nanoparticles and ease the electron transfer from the nanoparticles to SWCNTs. This consequently decreases the hole-carrier density of the p-type SWCNTs, resulting in the resistance increase. Owing to the direct nature and atom level resolution of this mechanism, the sensitivity is high and detection limit is extremely low.

**Figure 15.**
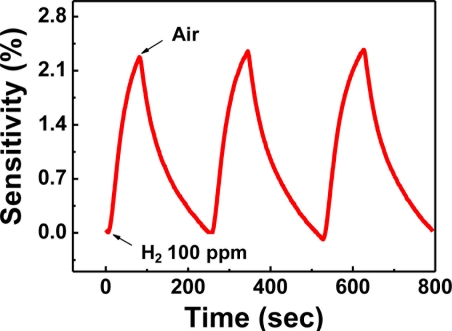
The real-time electrical response of sample 2 to 100 ppm H_2_ at room temperature.

As shown in Figure 15, the response curve of sample 2 is clear and the sensitivity reaches 2.3% at H_2_ concentration as low as 100 ppm, which is much higher than that of Pd nanowires. The detection limit of 10 ppm H_2_ in air was achieved using the sample 2.

## 6. Conclusions

Pd-based hydrogen sensing has been extensively investigated over the past four decades. More recently, the performance of hydrogen sensors has been greatly improved by the use of various Pd nanostructures, which was stimulated by the advancement of nanotechnology. Low-dimensional Pd nanostructures such as Pd thin films, Pd nanowires, and Pd nanoparticles have emerged to meet the requirement of fast, sensitive, and reliable detection of hydrogen gas. Although the nanostructures have many advantages, primarily due to the high surface-to-volume ratio, the manufacturability of hydrogen sensors based on them should be also considered for the practical use of the nanostructured sensors. Hydrogen sensors employing Pd nanostructures have unique attributes and issues that depend on the dimensionality of the nanostructures.

Two-dimensional Pd thin films suffer from structural deformations and hysteretic resistance behaviors although they are the easiest nanostructures to fabricate. These issues hinder the use of Pd films for sensing hydrogen reproducibly and in a scalable way. Use of a proper buffer layer or addition of small amount of second element to Pd can remedy the issues, accompanying the modulated properties such as decreased sensitivity and accelerated response. However, these modified Pd films are confronted with response time and hydrogen detection limit limitations. One-dimensional Pd nanowires show excellent sensor properties in aspects such as hydrogen-sensing scalability, response time, and hydrogen detection limit. However, these nanostructures are difficult to fabricate and their sensitivities fall short of those of Pd thin films. Pd nanoparticles can satisfy almost all the requirements such as high sensitivity, fast response, scalable detection of hydrogen, and low detection limit, if complementary structures are properly chosen.
